# An incremental training method with automated, extendable maze for training spatial behavioral tasks in rodents

**DOI:** 10.1038/s41598-019-48965-w

**Published:** 2019-08-29

**Authors:** Esther Holleman, Jan Mąka, Tim Schröder, Francesco Battaglia

**Affiliations:** Donders Institute for Brain, Cognition and Behaviour, Radboud University, Nijmegen, the Netherlands

**Keywords:** Long-term memory, Operant learning

## Abstract

We present a training procedure and maze equipped with sensors and automated feeders for training spatial behavioral tasks in rodents. The maze can be transformed from an enclosed box to a maze of variable dimensions. The modularity of the protocol and setup makes it highly flexible and suitable for training a wide variety of spatial tasks, and facilitates incremental training stages of increasing maze size for more efficient learning. The apparatus, in its software and hardware, is able to adapt to animal performance, adjusting task challenges and difficulty. Two different methods of automatic behavioral scoring are evaluated against manual methods. Sensors embedded in the maze provide information regarding the order of reward locations visited and the time between the activation of the cue via the nose-poke and the activation of the reward location sensors. The distributions of these reaction times differ between correct and incorrect trials, providing an index of behavior and motivation. The automated maze system allows the trainer to operate and monitor the task away from the experimental set-up, minimizing human interference and improving the reproducibility of the experiment. We show that our method succeeds in training a binary forced-choice task in rats.

## Introduction

Spatial behaviors are particularly developed in rodents as they have an innate drive to explore new environments and navigate through narrow passageways^1,2^. For this reason, many cognitive functions, including those that are not necessarily spatial in nature, such as working memory, are examined using tasks with a strong spatial component^3^. Place cells in the hippocampus are a useful model in the study of memory^4^. These neurons, among others, have spatially tuned responses, providing a neural read-out of behavior. Accurately assessing the spatial tuning of those cells requires large mazes, typically 80–100 cm or larger, rather than smaller, Skinner-box like setups, most convenient for e.g. operant conditioning training. The size of the maze does not present a problem for simple, exploratory tasks. However, shaping rodents into producing specific behavioral responses to cues can prove challenging in large environments that entice rodents to explore.

With setups typically used for behavioral electrophysiology, training these tasks is often labor-intensive, requiring the trainer to be near the maze to give cues, open and close barriers, and the reward the rat when it displays the desired behavior^5–7^. Furthermore, experimenter proximity and involvement can influence the performance of the task. The trainer may unintentionally signal the correct answer to the animal, as a result the animal may be responding to unintended cues of the trainer instead of the intended cue^8^. This is known as the experimenter-expectancy effect, and was first documented by Oskar Pfungst in 1907 in the case of the horse known as ‘Clever Hans’^9^. These possible confounds can make it more difficult and time intensive to train a task, and reduce the reproducibility of the task. These unwanted effects can be largely resolved through the use of automated training systems such as B.F. Skinner’s operant conditioning chamber^10^. These systems have evolved for various applications, and can include touch screens^11^, acoustically transparent chambers^12^ and high throughput systems implemented in the home cage^13,14^. The various custom made and commercially available automated training systems available are mostly implemented in Skinner boxes however. Automated systems designed for tasks in larger mazes are less commonly seen.

However, designing an automated system for a large maze is subject to different constraints than those posed by a Skinner box. The enclosed environment of a Skinner box provides an environment with minimal distractions. A similar effect could be achieved in a larger environment by implementing high, opaque walls around the track. This is not a possible solution for all tasks however, as the visibility of spatial cues is paramount for the formation of spatial representations, e.g. in the hippocampus. Another advantage of smaller training spaces such as Skinner’s operant chamber is that the cue can be immediately followed by a reward, facilitating association. In contrast, the likelihood of distracting events taking place between the cue-delivery and the discovery of the associated reward is increased significantly in a larger maze, because of the longer trial duration and the larger number of environmental stimuli, making it more difficult for it to make the association between the cue and the reward. In a large maze, these additional difficulties are exacerbated when all task components are trained simultaneously from the start of training. A task such as visiting a reward area associated with a particular cue tone consists of several subtasks that the animal must acquire. First the animal must learn to nose-poke to initiate a trial and receive the cue, learn that only one of the two reward areas can be visited in order to obtain a reward, and learn to return to the start-box and nose-poke to initiate the next trial, regardless of whether reward was received in the current trial. Attempting to train all these aspects at once in a large maze, where the animal can easily be distracted and the cue tone long forgotten before the distant reward area is reached needlessly increases the difficulty of the training process.

We propose a method to facilitate training complex tasks in a large environment by dividing the training into stages of increasing difficulty, and have created an automated setup that streamlines this procedure. Early training stages should minimize distractions and minimize the time between cue and reward to encourage association forming. The distance between the cue and reward can gradually be increased in each training stage until the final task to be trained is reached. Thus, in early training stages the reward location should be located close to the start-box where the cue is received to facilitate the development of an association between the two. When the response of the animal to the cue indicates the associations have been formed the distance between the cue and reward locations can incrementally be increased to increase the difficulty level of the task and the working memory load. The initial close proximity of the cue and reward locations also enable the creation of a more sheltered environment, decreasing the amount of possible distractions during early training stages for efficient learning.

Applying this gradual training schedule requires adapting the physical maze and the training software as the animal learns. To implement this method, we have created an automated training system consisting of a modular maze, hardware, and a software program to run training sessions. The modular nature of the maze facilitates incremental training stages of increasing maze size. At the beginning of each block of trials the software generates a randomized, counterbalanced trial schedule. Timing and delivery of cues is controlled by a micro-controller, receiving information from sensors located at relevant locations in the maze, and running each trial autonomously, with no involvement of the controlling computer. It is possible to override the decisions made by the micro-controller algorithm through the user interface of the software. Feeder units can be activated and trials can be canceled in this manner. This ability to intervene from a distance eliminates the need for the researcher to physically enter the experiment room and risk affecting the performance of the animal^15,16^. This system can be implemented on various maze designs for a variety of tasks. Possible modifications include adding a visual cue, visual feedback, or positioning two feeder units by the nose poke to provide cues in the form of flavored pellets and providing pellets of a flavor corresponding to the cue at the reward areas. The amount of reward areas can be increased through the addition of feeder units, for instance in the case of a radial maze with several arms. The system easily accommodates for the flexible placement of multi-modal cues and for different cue-reward schedules, therefore lending itself to a number of different experiments. We demonstrate its functionality here by training a tone-to-place association task (see e.g.^6^).

## Methods

### Animals and behavioral task

All animal procedures were approved by the Animal Ethics Committee of the Radboud University Nijmegen (RUDEC) and carried out in accordance to the Dutch guidelines and regulations for animal experiments.

Four six months old Long Evans male rats were housed in pairs and maintained on a reversed 24 h light/dark cycle at 85% of their ad libitum weight and trained on a two-alternative forced choice task to test working memory (see supplementary materials on animals and food restriction for details, Supplementary Fig. 9). Upon activation of the nose poke by the animal either a 7 Hz or 14 Hz tone was played as a cue to indicate which location would be rewarded when visited.

**Figure 1:**
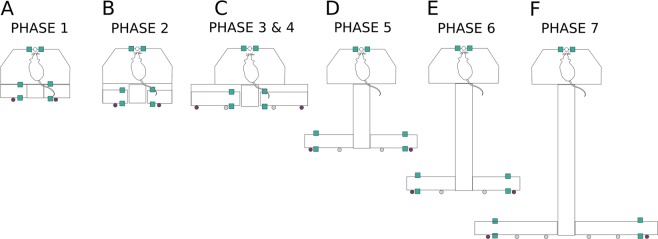
Configuration of the training environment at each phase. Locations of infrared sensor are shown as teal squares at the nose poke and the reward areas. Feeder locations are indicated by filled circles (empty circles represent possible feeder locations). (**A**) In phase 1, a small, enclosed space is created ensuring the cue and reward are administered in close proximity. (**B**) A gap between the arms of the maze and the start box platform is introduced in phase 2. (**C**) The reward area arms increase in length at phase 3. (**D**) At phase 5 the central arm is increased in length by 20 cm, the length of the reward area arms is increased to 20 cm. The feeder and sensor locations shift to the end of the reward area arms. (**E**) At phase 6, the length of the central arm is increased to 50 cm and at phase 7 to 80 cm (**F**). Also at this phase, the arms reach their maximal length of 40 cm and the distance between the central arm and the reward areas is increased further to 35 cm. Please note that although side arms were present in phases 5 to 7 these are not shown in this figure due to space constraints. Side arms were not utilized during trials (trials are only considered valid when the animal uses the central arm to reach the reward areas) and animals were trained to return to the start box via the central arm between trials. Animals spent only a small fraction of their total time in the maze during a block in the side arms (25^th^ percentile: 1.8%, 50^th^ percentile: 4.7%, 75^th^ percentile: 7.9%), which predominantly occurs at the beginning of a block while the animal briefly explores the maze prior to activating the first trial.

In the early phases of training the distance between the nose poke and the reward areas was minimized to encourage the formation of an association between the tones and reward areas. This distance was increased incrementally according to the performance of the animal, with the maze gradually morphing from an operant conditioning box into a full-sized maze (Fig. 1).

### Training protocol

Training began with habituation to the maze, followed by nose-poke training, automated feeder habituation, cue training, and ultimately the performance of the complete task in increasing levels of difficulty. To enable gradual acquisition of the different task components, training was divided into seven phases (Table 1; see General Training Description in the Supplementary Materials for an extended description of the training phases). The first four phases consisted of cue training, followed by three phases where the animals performed the complete task that included a working memory component due to the increased distance between the nose poke and reward areas. Each training session was divided into four blocks. The number of trials per block increased from 10 trials during the first phase, to 15 trials for phases two through five, 20 trials during phase six, and 25 trials in phase seven.

**Table 1:** Learning goals per training phase.

Overview of Training Phases	
Phase	Learning Goal/Desired Behavior
1	The task structure is acquired. Initialize trial through nose-poke, visit one of two reward areas, consume reward, and return to nose-poke for subsequent trial. Hint trials, where a pellet is dispensed at the reward areas associated with the tone, are used initially to encourage the animal to visit the reward area following a nose-poke. The time between the tone and the hint is gradually increased. When the animal displays any movement towards the correct area before the hint has been given three pellets are rewarded at that area.
2	Respond to the cue tone through head movement in the direction of the associated reward area.
3	Respond to the cue tone with complete body turn towards associated reward area.
4	Respond to the cue tone with complete body turn and forward movement towards associated reward area.
**5**	Maze arms moved back 20 cm from start-box. From this phase onwards the animal must respond to the cue tone by moving towards one reward area through the central arm and waiting at reward area for the reward.
**6**	Maze arms moved back 50 cm from start-box.
**7**	Maze arms moved back 80 cm from start-box.

In all phases the animals independently returned to the start box upon completion of a trial. Animals were able to initiate a new trial at the nose poke immediately following the end of the previous trial. The length of the inter-trial interval therefore depends on the speed at which the animal returns to the start box and activates the nose-poke sensors. Inter-trial intervals for all phases are shown in Supplementary Fig. 11.

### Randomization for sequence of rewarded areas within a block of trials

A sequence of random integers (0 or 1) was generated to determine which side to cue/reward in each trial. Sequences with consecutive repetition of a particular side for more than 3 trials were discarded to prevent the animals from developing a bias towards a particular side. Similarly, sequences with frequent switches of rewarded sides between trials were also removed to reduce the natural tendency of the animal to alternate between reward sites with each trial^17^. This alternation strategy commonly applied by rodents may reflect natural foraging behavior where it is not strategic for the animal to return to a depleted food source^18^. See supplementary material for a full description of the algorithm.

The randomization algorithm was tested by generating the sequence of cues for a block of trials ten thousand times. For each generated sequence several measures were tested such as the percentage of left versus right rewarded trials, the amount of alternation between sides, the ratio of left to right transitions, and the ratio of right to left transitions (Supplementary Fig. 6). If any patterns were found that could potentially encourage a bias the algorithm was altered until no potential biases were present. We found, however, that a ratio of 40% alternation transitions to 60% same side transitions was most conducive to learning as the alternation tendency of the rodents was sufficiently strong that it must be actively discouraged by presenting the animal with more same-side transitions than alternation transitions. To study spatial memory in animals it is imperative to ensure that the behavioral readouts can be attributed to spatial associations to the cue and are not the result of other underlying strategies that produce similar behavioral readouts^19^. For example, rats are excellent in identifying patterns in the randomization sequence in order to predict which side has a high probability of delivering reward in the upcoming trial, given the outcomes of the previous trials^20^. To ensure animals could not acquire too many rewards by using a strategy other than the task to be learned, simulations using various strategies were performed on the sequences of trials generated with the randomization algorithm. Both simple strategies such as consistently choosing one side over the other or spontaneous alternation between reward areas as more responsive strategies such as selecting the reward location opposite to the one rewarded in the previous trial (win-shift) or returning the location rewarded in the previous trial (win-stay) were tested. The sequence generator was deemed sufficient when the strategy simulations could not score above 60% on average over any of the session lengths used in the experiment.

### Analysis methods

**Figure 2:**
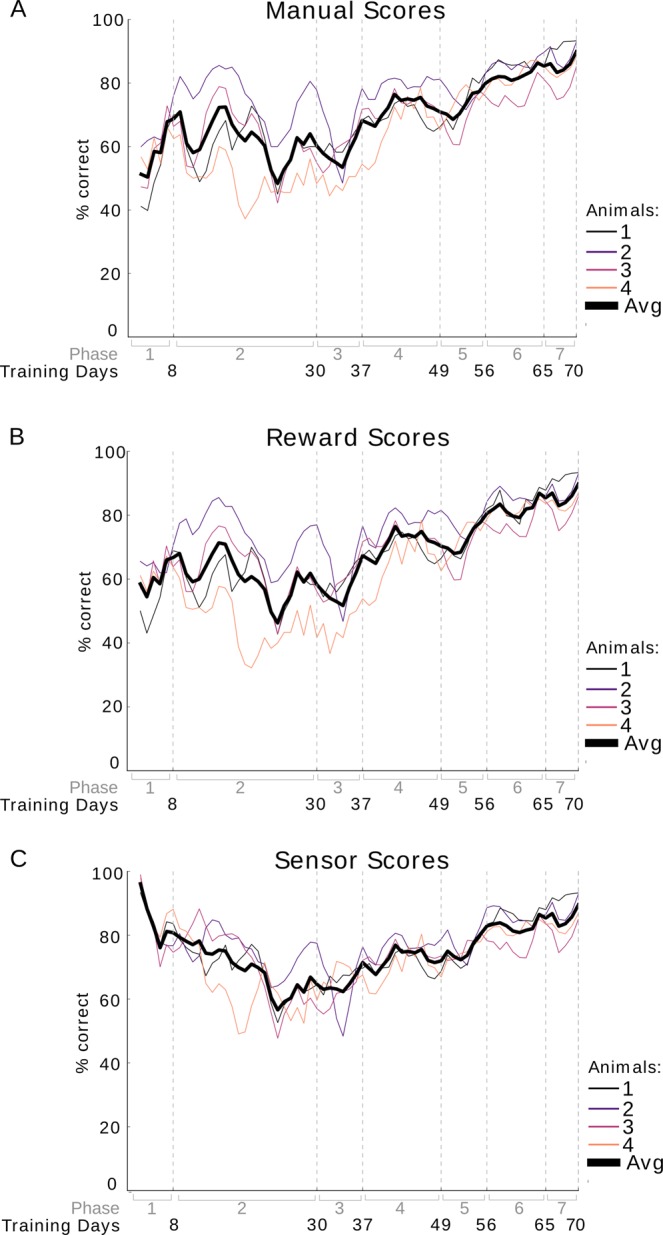
Comparison of scores calculated manually during training, scores based on the number of rewarded pellets, and scores as recorded by the sensors. A rolling average over 3 days was used to calculate the percent of correct scores over the training days (training phase shown in gray). The scores for each animal are plotted separately and also as an average over all animals (thick black line). Scores based purely on sensor readings are unreliable for the first four phases as the tail of the rat could activate the sensors unintentionally. Additionally on the first two days of the first phase reward automatically followed the cue in many trials. These hint trials were excluded from the scoring process however they do occur in the sensor data, resulting in unusually high sensor scores in the first phase. (**A**) Results from manual scoring. (**B**) Scoring based on amount of rewards given to the animal. In phase 1, the criteria for a correct trial was set at three or more rewarded pellets, in phase 2 to 5, two or more pellets, and in phase 6 and 7 one or more pellets. (**C**) Scores based on sensor data from sensors at the reward locations. Activation of a sensor at the reward area corresponding to the cue resulted in a correct trial.

The learning curve in Fig. 2A was acquired using manual scoring to mark a trial as correct or incorrect. Figure 2B shows the scoring based on the amount of pellets rewarded per trial, and the scoring in Fig. 2C is based on sensor data. A rolling average over 3 days was used to calculate the percent of correct scores over the training days. The scores for each animal are shown separately and also as an average over all animals.

**Figure 3:**
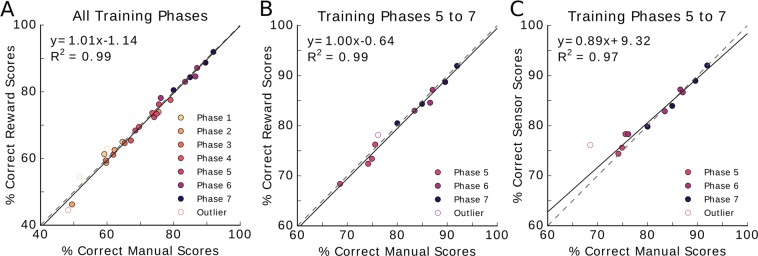
Scoring methods compared in scatter plot. Each phase contains four data points, representing the average score obtained during the specified phase for each of the four animals. Phases are depicted in different colors. Outliers, indicated by open circles, were removed for the regression analysis (see Methods). The dashed diagonal line indicates the unity line, where both scores are identical. The solid line represents the linear regression fit through the data points. (**A**) The percent correct for manual scores are plotted against the percent correct for the reward-based scores. All data points lie close to or on the unity line indicating the similarity of these scoring methods (slope = 1.01, y-intercept = −1.14%, R^2^ = 0.99, p = 2.03 × 10^−25^). (**B**) Reward scores as a function of manual scores for phases 5 through 7 (slope = 1.00, y-intercept = −0.64%, R^2^ = 0.99, p = 1.26 × 10^−9^). (**C**) Sensor scores as a function of manual scores for phases 5 through 7 (slope = 0.89, y-intercept = 9.32%, R^2^ = 0.97, p = 1.82 × 10^−8^).

For scoring method analyses shown in Fig. 3, outliers were removed by first performing a linear regression and calculating the standard deviation of the absolute value of the distances between the data points and the regression fit. Data points located further than 3 times the standard deviation away from the regression fit were marked as outliers. Subsequently, another regression fit was performed through the remaining points. This second fit is shown in the subplots, outliers are displayed as open circles.

Timed-out trials were scored as incorrect, however they were not included in the reaction time density distributions as they are all set at the predetermined threshold, for example at six seconds, and therefore not representative of actual animal behavior. Also, due to the inaccurate sensor readings in earlier phases only reaction times from the full task, phases five to seven were included.

**Figure 4:**
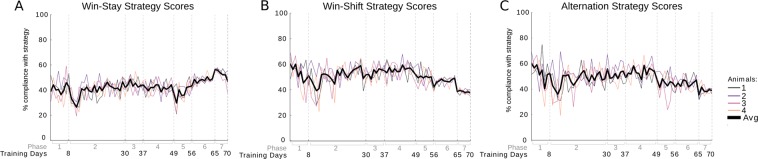
Animal responses compared to strategy responses. Percent compliance to strategy (y-axis) over training days (x-axis, phases indicated in grey). (**A**) Responses for each animal compared to expected responses when applying a win-stay strategy. (**B**) Animal responses compared to win-shift strategy responses. (**C**) Animal responses compared responses expected for spontaneous alternation.

Learning was assessed through automatically calculated scores. These scores were compared to manual scores throughout training to ensure accuracy. To determine if correct scores were due to animals behaving according to the task or according to a strategy, scores were tested for compliance with common strategies such as win-shift, win-stay, and spontaneous alternation strategies (Fig. 4).

More detailed accounts can be found in the Supplementary Materials for the training protocols (Supplementary Table 1, Supplementary Fig. 1), the randomization testing (Supplementary Fig. 6), strategy simulations (Supplementary Figs 7 and 8) and compliance of animal responses to strategy responses (Animal Scores on Use of Strategy in the Supplementary Materials).

## Results

The learning curve of the animals over the different phases is shown in Fig. 2A. The percentage of correct trials is shown on the y-axis and the training days on the x-axis, where the phase in which the training day occurred is also displayed. An increase in performance over phases can be observed. The average score across animals increased gradually from 59% correct in the first phase to 87% correct in the last phase.

### Comparison of scoring methods

To test the accuracy of the automated scoring, scores were manually recorded during training (Fig. 2A). Two methods of automated scoring were compared, scoring based on the number of rewards given (Fig. 2B) and scoring based on sensor information (Fig. 2C). As described previously, training this task involved rewarding based on performance. The first and second phase of the cue training included ‘hint’ trials where one reward was given immediately following the cue tone. In the manual scoring, hint trials were excluded. However, as the animal often did move to the correct reward area following a hint, the sensors record these trials as correct. The reward scoring does not count hint trials as correct, as only one pellet is delivered during these trials compared to several pellets in trials where the animal chose the correct location without the help of a hint. With the reward scoring method a trial was marked as correct depending on the amount of reward given. The amount of rewards necessary to qualify a trial as correct depended on the training phase. In phase 1, the threshold for a trial to be considered correct was set at three or more rewarded pellets. In phase 2 to 5, two or more pellets, and in phase 6 and 7 one or more pellets.

The similarity between the learning curves of the manual scoring method and of the reward scoring method (Fig. 2A,B) indicates that reward scoring is a suitable replacement for manual scoring. Linear regression analysis also supports this, as the fit has a slope of 1.01, indicating the results for manual and sensor scoring methods are close to identical (Fig. 3A, R^2^ = 0.99, offset −1.14%, p = 2.03 × 10^−25^).

From phase five onwards, both manual and reward scoring methods (Fig. 3B, R^2^ = 0.99, p = 1.26 × 10^−9^) as well as manual and sensor scoring methods are highly correlated (Fig. 3C, R^2^ = 0.97, p = 1.82 × 10^−8^). In both cases the data scattered around the unity line with slopes of 1.00 and 0.89 respectively, and offsets of −0.64% and 9.32%, respectively.

### Animal behavioral responses in light of common strategies

Animal responses to the cue were compared to strategies commonly applied by rodents in binary choice tasks to ensure animals learned the task intended to be taught. Given the response of the animal to the first cue in a block of subsequent trials the response for the remaining trials was predicted for several common strategies. These strategy responses were then compared to the actual responses of the animal (Fig. 4). This comparison is described in more detail in the Animal Scores on Use of Strategy section of the Supplementary Materials.

### Reaction time distributions

**Figure 5:**
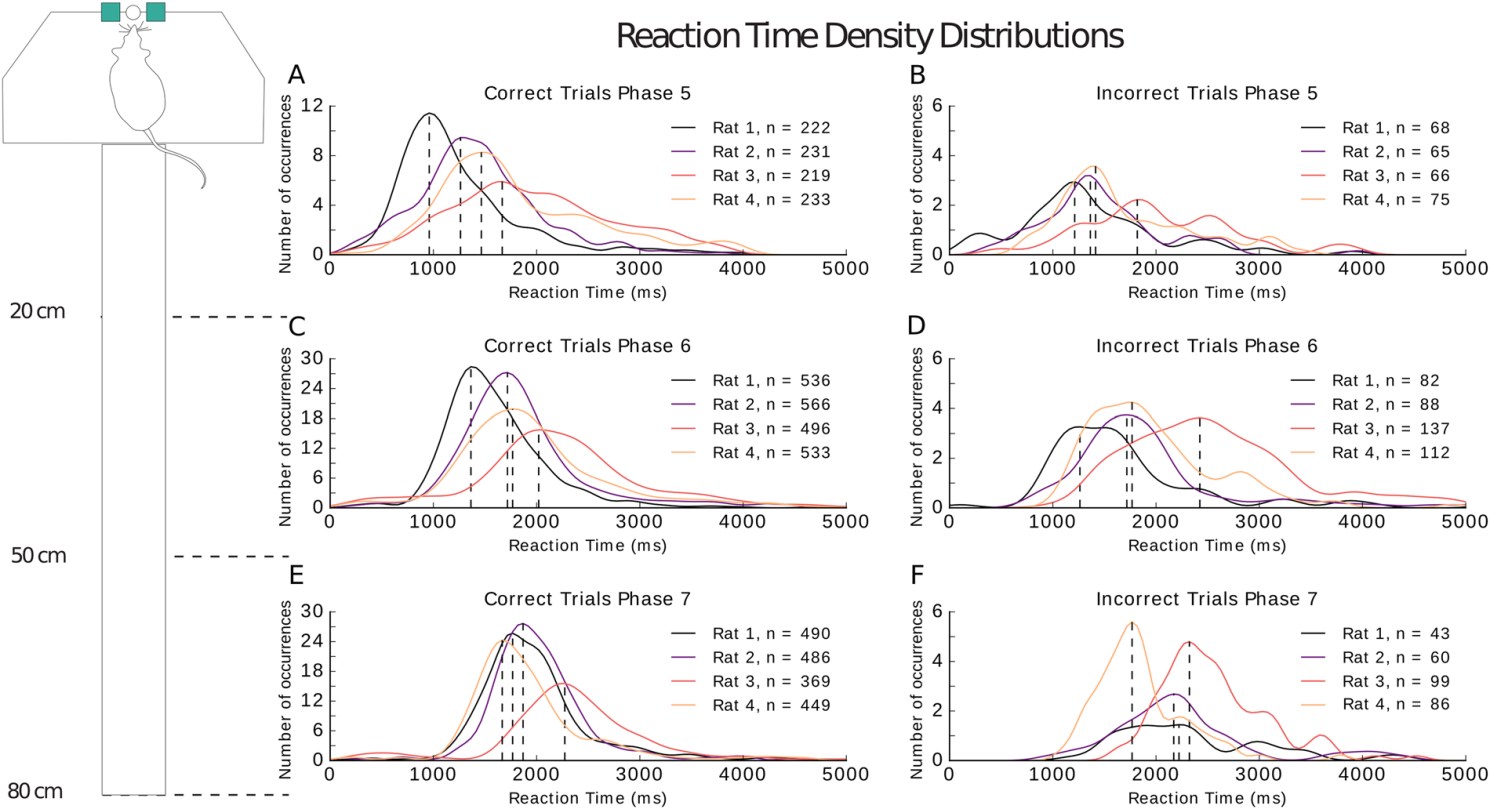
Reaction time density distributions for the full task (phases 5 to 7). Distributions are plotted per rat. The number of trials included in each distribution are noted in the legend. Reaction time is shown in milliseconds. Correct trials are plotted in the left column (**A**,**C**,**E**) and incorrect trials in the right column (**B**,**D**,**F**). Each row represents a phase. The dotted lines are the modes for each distribution.

A trial starts when the nose-poke sensors are activated and ends either when the rat crosses the sensors at one of the reward areas. A trial is also terminated when the maximum trial time is reached before reward sensors are activated. The thresholds for this time-out differ per phase. In the first phase, the animal has six seconds to make his way to the reward areas, as the difficulty increases with each phase the time allowed per trial decreases. The distributions in Fig. 5 show the reaction times for each animal for both correct and incorrect trials (left to right) recorded in phases five through seven (top to bottom). The number of trials included in each distribution is noted per rat.

From the shape of the distribution information about the behavior of the animal may be inferred. A broad curve with widely varying reaction times indicates inconsistent behavior. Slow reaction times were often due either to hesitation following the administration of the cue or distracted behavior where the animal was clearly disinterested in the task and likely no longer motivated by the food reward. In those trials, animals only performed at chance level. An unusually fast response points towards the animal following a non-task-related strategy, for instance the animal may have based its choice on the result of the previous trial and therefore had no need to attend to the cue. The reaction time distribution of all incorrect trials spans over a large range of values, as it covers the fast reaction times, likely reflecting the use of a strategy, to the slow reaction times presumably related to indecision, virtual trial-and-error behavior^21^, or lack of motivation. In comparison, distributions of correct trials often cover a much smaller range of reaction times, resulting in a narrower distribution. Reaction times of animals that have successfully made the association between the cue and the reward areas vary between 1000 and 2000 milliseconds. This average increases with the size of the maze, as the distance between the nose-poke and the reward areas increases.

**Figure 6:**
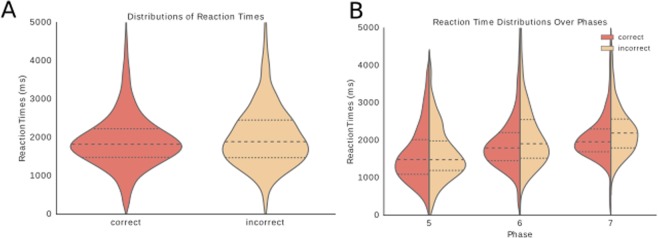
Comparison of correct and incorrect reaction time distributions. (**A**) Distributions of reaction times in milliseconds for phases 5 to 7 plotted together to compare correct trials to incorrect trials (p = 1.24 × 10^−7^, two-sample Kolmogorov-Smirnov test). (**B**) Reaction time distributions for each phase separately with reaction times in milliseconds plotted on the y-axis and phases on the x-axis. The left half of the violin plots represent the reaction times of the correct trials and the right side those of the incorrect trials. Reaction times increase over phases as the distance travelled increases with the expansion of the central arm of the maze.

The differences between the correct and incorrect reaction times increase as the phase of training progresses (phase 5: p = 0.16, phase 6: p = 0.0001, phase 7: p = 7.2 × 10^−9^ two-sample Kolmogorov-Smirnov test). In Fig. 5A the reaction times of all trials in phases 5 through 7 show a significant difference between incorrect and correct trials (p = 1.24 × 10^−7^ two-sample Kolmogorov-Smirnov test, interquartile range correct trials = 558 ms, incorrect trials = 890 ms). Figure 6B shows that the distributions for the correct trials narrow with each successive phase. The interquartile range for correct trials is 840 ms in phase 5, 650 ms in phase 6, and 520 ms in phase 7. For the incorrect trials this effect is not present (phase 5: 765 ms, phase 6: 746 ms, phase 7: 669 ms).

## Discussion

We have presented a novel training procedure and setup for maze-based tasks. The modular extension of the training apparatus enables incremental training phases of increasing difficulty. The setup is automated, with the within-trial logic controlled by a micro-controller, with the overall experimental logic overseen by a PC, enabling user intervention when needed. Micro-controller based automated mazes and training setups have recently been proposed^22–24^. Here we propose a modular approach, with “slave” micro-controllers handling sensors in each maze component and a “master” component coordinating the trial management, enabling the easy extension and customization of the environment. All components are low-cost and readily available, and assemblage of the system is not difficult.

We have shown that rats can learn the sound-to-place association task when trained through this system. The first phases, which consisted of learning the associations between the cues and the reward areas were the most time consuming, and took place in the reduced-size version of the setup. The gap created when the arms are moved away from the start box only appeared to affect the behavior of the animals when first introduced in the second phase. Once the animals were accustomed to the gap between the maze arms and the start-box moving the arms further away incrementally did not affect their performance. Furthermore, none of the distances used (up to 80 cm) appeared problematic for working memory. Rats were able to complete 100 trials a day with ease when the number of trials was increased gradually during training. Notably, this was based on appetitive food rewards, whereas other approaches to obtaining large number of training trials involved invasive stimulation of reward structures (e.g. the medial forebrain bundle) for reward^25^. Choosing a task that accommodates a large number of trials per day allows for the acquisition of a sufficient amount of behavioral data for statistical analysis and is beneficial for electrophysiological data acquisition. The automated training system can be implemented in many different tasks and mazes. It is also possible to work without a cue and simply provide a reward at a particular area in the maze in which the sensors have been activated. The amount of different cues and reward areas can be extended through the addition of more feeder and sensor units. Variations of cues and rewards are also possible. For instance, feeders can be attached to the nose poke device to feed different flavors of pellets as cues instead of the cue tones used in this experiment. The feeders at each reward area can also contain different flavors to match the cue flavor. Possible extensions of the setup include the addition of an odor distributor to provide cues, or a display to provide visual cues.

Bower and McNaughton show that training the same task with different methods resulted in differential hippocampal encoding^5^. Rats were trained to remember sequences of reward locations containing repeated locations within different sequences in a delayed alternation task on a continuous T-maze. For instance, location “b” (the central arm of the T-maze) is present in both the sequences “a-b-c” and “d-b-e”. The rat must differentiate between the two contexts in order to decide which location to visit after location “b”. When animals were trained, as an intermediate step, by also placing reward at the end of the repeated segment, they learned the sequences correctly, yet hippocampal ensemble activity did not differentiate the sequential context of the repeated segment. In rats that learned the task with the help of moveable barriers that directed their path to the reward areas hippocampal activity did differentiate between the different sequential contexts. The method proposed here increases maze size and trial duration, while leaving the structure of the task unchanged. We think that this may be advantageous in the study of neural representations of rule learning and task structure.

An automated training system lends itself well to examining the effects of various training methods on neuronal activity as it offers a controlled way to train these different methods, without interference. For examples, comparisons between various forms of cueing could be examined, such as cue LEDs located at the reward areas, or cue LEDs on either side of the nose-poke device, indicating which side will be rewarded. Alternatively, the same working memory task described here could be trained with and without barriers placed in the maze, directing the animals to the correct reward location. This requires a system where a task can be trained both with and without barriers such as automated doors. However, most automated mazes employ doors to enforce trial structure, for example to restrain the animal in the start-box before the initiation of a trial, to ensure it does not visit multiple reward areas after an incorrect behavioral response, and to direct the animal back to the start-box^14,26^. We show here that it is possible to train rats to both conform to the trial structure and perform the desired task through incremental training phases without barriers, thereby facilitating experiments where the use of barriers may influence the neural processes to be studied, yet automation of the trial structure is desired.

Several measures can be taken to improve the experimental set up described in this paper. These include creating more consistency among phases by introducing a gap between the start-box and the maze arms during habituation and increasing the amount of training sessions to two per day in all phases to expedite the learning process and improve the accuracy of the analysis. Although block length should remain short in the first phase to provide enough time for the animals to habituate to their new routine in a stress-free manner, increasing the amount of blocks of trials per day could significantly accelerate the learning process and decrease the amount of training days necessary, provided enough breaks are present between blocks. In this experiment, rats were trained in half-day sessions for the first five phases. Experience in later stages showed no loss of focus, motivation, or performance in the animals when two training sessions were provided daily, one morning and one afternoon session, with a break of at least two hours in between, allowing the animals to rest, drink, and regain motivation. Another benefit of increasing the amount of trials per day is that it allows for feeding the animals their daily amount of food required to maintain a healthy weight within the task and avoids the necessity to feed extra in the cages after training sessions. This circumvents problems with dominance between animals housed together^27,28^, where the dominant animals consume more food in the cage and perform poorly as a result due to lack of motivation, while the performance of non-dominant animals suffers from anxious behavior during the task as a result of underfeeding. Currently, in the first three phases the maze arms are placed close to the start-box. This, however, encourages the rats to take a shortcut to the reward areas. Observations from this experiment indicate that it would be more beneficial to move the arms a short distance away from the start-box during habituation. This will provide the animals with more time to become acquainted with the gap between the arms and the start-box during the habituation period instead of between training phases. As a result the performance of the rat will be less influenced by transitions between phases. Moving the arms a short distance from the start-box initially will also require the animals to travel via the central arm to arrive at the reward locations for all phases of training instead of only in the later phases. This creates consistency across phases that will ease the interpretation of neural data and enable automation of the entire task, as sensor data would be reliable from the first phase onwards, improving the quality of the analysis.

In conclusion, we presented a novel framework for animal training in spatial, maze-based task, which may prove useful for those attempting to train animals in the large setups that are needed for example for neural ensemble recording experiments.

## Supplementary information

Supplementary materials
