# Supplementary material for: An incremental training method with automated, extendable maze for training spatial behavioral tasks in rodents
Source: Sci Rep. 2019 Aug 29;9:12589. doi: 10.1038/s41598-019-48965-w (PMC6715809; doi:10.1038/s41598-019-48965-w)
Supplement: Supplementary file 1 — Supplementary materials [file 41598_2019_48965_MOESM1_ESM.pdf]

Supplementary Materials

An incremental training method with automated, extendable maze for training spatial behavioral tasks in rodents

Esther Holleman, Jan Maka, Tim Schröder, Francesco Battaglia
Donders Institute for Brain Cognition, and Behaviour, Radboud University
Email: estherholleman@gmail.com, f.battaglia@donders.ru.nl

General training description

The desired behavior to be trained in this task involves the presentation of a cue tone, either 7 or 14 kHz, in response to which the rat must move to the location associated with the tone. The 7 kHz tone signals that the rat must move to the left reward location, in the case of a 14 kHz cue it must move to the right reward location. When the rat moves to the correct location pellets will be delivered at the chosen reward location. If the rat chooses incorrectly, no reward will be provided. If the rat chooses the incorrect location first and immediately proceeds to the correct location afterwards it will not be rewarded as the task dictates that the correct location must be visited first. This is implemented through the use of sensors in the maze. The pair of sensors activated first will report their activation to the main program and prevent any other sensor activation from triggering a reward.

At the core of this task lies the association between the cues and the reward areas. The rat must learn that tone played at the beginning of each trial, initiated by a nose poke, relates to a reward from a particular reward location. Furthermore, the rat should also move to the reward location in response to the cue in order to receive the reward at that location. A delay between the stimulus presentation and the choice leading to a reward can be used to test working memory. However, efficient initial training for this behavior requires a short amount of time between the cue and the reward for an association to successfully be made between the two.

The modular structure of the maze due to its adjustable arms enables the successive expansion of the maze to larger sizes. The reward area arms can be moved increasingly further away from the nose poke, where the rat receives the cue. Initially these arms connect to the start box platform, minimizing the distance and therefore the time, between the cue and the reward. The start box and perimeter of the maze are equipped with 40 cm high walls to limit distractions from external stimuli.

The task structure is established during the first training phases. Advancing to the next phase requires that the animals have shown capability in following the task structure, always nose-poking first, moving to a reward area, consuming pellets at reward and

returning to the nose-poke. The nose-poke sensors are available to trigger a new trial immediately following the completion of the previous trial. The length of the inter-trial interval depends on the speed at which the animal returns to the start box and activates the nose-poke sensors.

At the next stage the association between the cue, the desired behavior, and the reward must be established. Compared to later phases this phase is the most time consuming (29 days, see Table 1 for a comparison with other phases). In order to build a strong association the animal must actively choose a side in response to the tone in order to receive a reward. In this phase the animal must always first move towards the cued side. At first a reward will follow after only a small movement of the head towards the correct reward location, the required movement to trigger reward will become increasingly larger, leading up to the point where the animal must move to the reward location and wait there before receiving a reward.

Once an average performance of 70% correct is achieved over at least 3 training days the maze configuration can be adjusted by moving the arms further away (see Supplementary Figure 1) from the start box. The rat should then be trained in the same manner, continuing the same task as before in the new configuration, until performance is satisfactory.

The maze arms, and therefore reward locations can be moved increasingly further away gradually, each time ensuring the animal achieves a consistently sufficient performance before proceeding to the next phase. With the movement of the reward arms the point at which the animal is able to directly approach the reward areas will also be moved increasingly further away from the cue. Whereas at first animals could turn directly towards one location or the other following the cue, they must now walk down a central arm for an increasingly large distance before committing to a choice. In this task configuration, choosing the correct location requires holding the location associated with the cue in working memory. This will be trained in several phases where the distance for the rat to hold the cue in memory will be increased incrementally with every phase.

Below, we provide a detailed protocol for training:

1. Habituation to maze & the association of reward locations with reward

1. Habituate animals to the experimenter and to being picked up and placed back into the home cage, until this produces no apparent stress.
2. Begin the habituation to the training environment by placing group-housed, food restricted animals in the maze together for a short period of no longer than 2 minutes with pellets available at reward areas and at the edge of the nose poke tube. Following a break of 5 minutes in the home cage the rats can be placed back in the maze and left there for one minute longer than the previous session. Repeat this process until rats can comfortably be in the maze together for 6 minutes. Moving back and forth from home cage to maze will also accustom the animals to

the process of entering and exiting the training environment. The goal of these sessions is to generate an association between the training environment and food reward. If at any time an animal displays signs of distress, remove the animals from the maze, provide a 5-minute break, and repeat the previous session until the animals can comfortably remain in the maze for the desired time. Due to a neophobia towards food found in novel environments the animals may not eat the food on the first day [1].

3. Repeat this process the following day, leaving the animals in the maze together for 5 minutes initially, followed by a 5-minute break in the home cage. Repeat this process further while incrementing the time in the maze by one minute per session.
4. The following two days repeat the above, starting with an in-maze time of 2 minutes on the first day, with one animal at time to accustom the animals to spending time in the maze alone for increasingly longer periods of time. On the second day begin with a 5-minute session.
5. Only move an animal on to the nose poke training when it consumes the pellets provided in the training environment. If this is not the case after the fourth day repeat the 5-minute sessions until the animal is comfortable eating in the maze. Growth curves should be carefully monitored and the optimal percentage of normal weight for each animal identified and maintained throughout the duration of the training.

2. Nose-poke training

Place a pellet near the edge of the nose-poke tube. Wait until the rat consumes this pellet. Repeat this several times before placing the pellet slightly farther up the nose poke tube. Gradually increase the distance of the pellet from the entrance of the nose poke tube until the animal is nose poking far enough into the tube to activate the sensors.

Once the rat displays nose-poking behavior correctly in a consistent manner, follow the nose-poke reward immediately with a reward at one of the reward areas. Do not reward in the nose-poke again until the rat has visited the rewarded location. Randomize the locations rewarded in such a way that the rat visits both equally often (see 'Randomization' section for details). The rat must always nose-poke before receiving a reward at a reward area. Bait the nose poke for 5 trials, and follow with food at a reward area each time, before returning the animal to the home cage. Repeat, while increasing block size by one trail each time, until animals reach 10 trials per block.

3. Automated feeder habituation

The rat is still rewarded with a pellet for nose-poking, however the nose-poke is now followed by delivery of food at a reward location via the automatic feeders. The rat will need to habituate to the sound of the feeders. Phase out food at the nose-poke slowly until the rat is nose-poking and receiving the resulting reward at the reward areas only. Initially a home cage break should be provided after 5 consecutive trials. The amount of trials should be gradually increased to 10 trials. Feeder habituation is complete when the animal has habituated to the sound of the automatic feeders and behaves according to

the task structure. It must nose poke to initiate a trial, immediately proceed to the active feeder, consume the reward, and return to the nose poke location to initiate the next trial for at least 10 trials within a session.

4. Cue Training

Blocks initially consist of 10 trials per block. A session consists of 4 blocks.

Phase 1

1. A tone (randomly selected at each trial from two possibilities) is played in response to nose-poke. Reward the animal with 2 pellets at reward area immediately following the nose poke. Doing this repeatedly allows the rat to become accustomed to the sound and builds the association of sound with reward.
2. The following day, maintain the same trial structure as above, however incorporate a 2 second delay after the nose poke. If the rat reacts by moving towards the correct area reward with 4 pellets. Activate the feeder as soon as the rat makes any motion, however small, towards the correct area. Other behavior results in a reward with one pellet at the correct reward area immediately following the 2-second delay.

Phase 2

3. Increase block length to 15 trials.
4. On the first day of phase two reward automatically for nose poke on the first trial, however for half of the remaining trials (randomly chosen) do not automatically reward for nose poke but wait until the rat moves in one direction or the other. Ignore any movement towards the incorrect reward area, and end the trial if the animal turns towards the incorrect area completely. Immediately reward any movement toward the correct reward location with 4 pellets.
5. No longer reward automatically on the second day. Movement towards the correct reward location, minimally a 10 to 30 degrees head-turn should be rewarded with 3 pellets in the cued location. If the animal does not move or moves more than 45 degrees towards the non-cued reward location do not reward and wait until the trial expires after 5 seconds. The nose-poke will then be available again for the next trial. Move to step 6 when the animal responds to the tone with the desired behavior of a 10 to 30 degree head turn in at least 70% of the trials during a session, for at least 3 sessions.
6. Head movement of minimally 60 degrees results in reward. This response must be present in 70% of the trials within a session before advancing to the next step.
7. More head movement (60 to 90 degrees), and/or some body movement is required (shoulders should turn towards correct reward location). Again, this response must be present in 70% of the trials within a session before advancing to the third phase.

Phase 3

8. Rat should be facing correct reward location before reward is given. Advance to Phase 4 when this response is present in 70% or more trials within a session.

Phase 4

9. At this stage the animal should not only be facing the correct reward location but

also show movement towards this location before reward is given. Advance to Phase 5 when this response is present in 70% or more trials within a session.

5. Full Task

A phase is completed when the animal reaches an average of 70% correct.

Phase 5

Expand maze, move arms backwards 20 cm so that rats must take the central arm to reach the reward.

Phase 6

Move the arms back another 50cm from the start-box and increase block length to 20 trials.

Phase 7

Move the arms 80 cm from the start-box and increase the block length to 25 trials.

Experiment Design

		Central Arm Length (cm)	Trials Per Day	Time Limit (s)	Min. Reward (num. pellets)	R+ time limit (s)	R+ (num. pellets)	R++ time limit (s)	R++ (num. pellets)
**Cue Training**	Phase 1	0	30	6	2	5	4	3	5
	Phase 2	5	60	6	1	5	2	3	3
	Phase 3	5	60	5	1	4	2	3	3
	Phase 4	5	60	5	1	4	2	3	3
**Full Task**	Phase 5	20	60	6	1	4	2	3	3
	Phase 6	50	80	6	1	4	2	3	3
	Phase 7	80	100	6	1	4	2	3	3

Supplementary Table 1: Settings for each phase defining the length of the central arm in centimeters, number of trials per day, time allowed (in seconds) for a trial, minimum number of pellets to be rewarded for a correct answer, and the timing thresholds number of extra pellets dispensed for response windows rewarding faster reaction times.

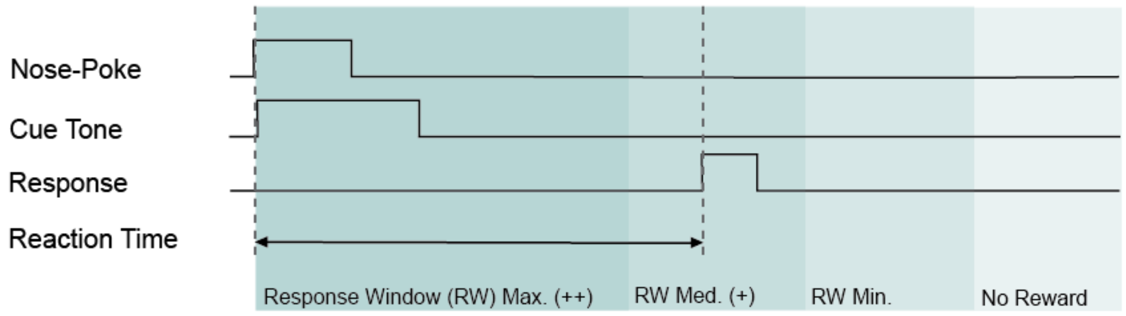

Supplementary Figure 1: Trial structure for cue training (response window dependent reward from Phase 3 onwards) and the full task. A trial begins with a nose poke, which triggers the playing of a cue tone for 1 second. The reaction time is measured from the onset of the cue tone to the activation of the sensors at the reward location. Several response windows are defined that determine the number of pellets dispensed, encouraging the animals to respond to the cue in a timely manner. Faster responses are rewarded more than slower responses. If the animal responds slower than the time allowed no reward is given.

Technical Implementation

We created an automated training system consisting of the following modules:

- **Input devices** to register the actions of the animal. We used infrared (IR) sensors. When the animal crosses the infrared beam this action is translated into a voltage change that signals to the controllers that the sensor has been triggered.
- **Interaction (output) devices.** In our case these were pellet dispensers referred to as feeders.
- **The main controller** that enables basic communication between registration and interaction units. It also sends and receives data from the computer running the software.
- **A software program** that manages the training sessions and allows interventions such as the cancellations of trials or the activation of the feeders to deliver reward to the animal for desired behavior not detected or rewarded successfully in an automatic manner.

Every module has its own unique task and is independent from the other modules. For instance the feeder unit checks its own performance continuously when activated, namely if it has dispensed the amount of pellets specified, and adjusts its actions accordingly. This reduces the probability of errors occurring, is useful for debugging, and ensures modules can be easily modified and improved without affecting the rest of the system.

The prototype of this system consisted of Arduino Uno microcontrollers functioning as the motherboard an IR sensor board, and Arduino Nano microcontrollers to control the feeder units. This construction allowed for fast prototyping. A future version of the system could replace the Arduino boards of the slave microcontrollers with custom designed, printed circuit boards equipped with microcontroller chips.

Hardware and Software Components

Main Controller

The main controller is an essential element of our system. We used an Arduino Uno as the master in the I2C protocol. Importantly, the experimental trial logic is handled on the main controller. This provides a speed advantage, as the communication between the master and slave microcontrollers using I2C is much faster than the serial port communication with the computer. The Master microcontroller can be accessed and controlled by the user via a software interface on the computer. This allows for interventions during a trial such as ending a trial or block earlier than planned or to provide additional reward where this may be beneficial or necessary to motivate the animal. I2C extenders provide the ability to connect the master controller to a large number of slave units.

IR Sensor Board

The sensor board consists of an Arduino Uno microcontroller with infrared (IR) sensors connected to its digital input pins. When the main controller is waiting for sensor input it requests information regarding sensors status ten times per second from the sensor board. Each sensor pair is identified with a number. Once a sensor is triggered the sensor board saves the number assigned to that sensor. This variable cannot be overwritten until the next request from the main controller, during which the number of the activated sensor is sent to the main controller and the variable is reinitialized. This ensures only one sensor reading is received by the main controller per request. When the main controller receives the number of the triggered sensor, it closes the communication with sensor board and the trial ends.

Feeder

Each feeder is an independent slave device, driven by a microcontroller that receives, via I2C protocol, the number of pellets to release. The feeder device consists of a 3D printed pellet reservoir containing a rotating disc, a servomotor and an electronic circuit with IR sensor, I2C extender and microcontroller. The program of the device independently controls the fulfillment of tasks as follows:

1. The feeder microcontroller receives the number of food pellets to dispense.
2. The feeder microcontroller triggers the servomotor to spin the disk.
3. IR sensors, located in the tube through which the pellets reach the reward area, register the number of pellets successfully dispensed.
4. When the sensors in fail to register any dispensed pellets the servomotor spins the disk in the opposite direction in an attempt to dislodge any obstructions.
5. When the number of registered pellets reaches the specified amount, the servomotor ceases its activity.
6. The feeder microcontroller sends information to the master microcontroller (main controller) that the pellets have been successfully dispensed.

Software Program

A Python program with a graphical user interface (GUI) is used to run the experiment. The core of this program is the Experiment class, which defines all parameters and functions related to a specific experiment. It is also responsible for storing data. The second layer is GUI interface, which enables the trainer to intervene during an experiment and review the results gathered. The software was developed in Python as this programming language allows for rapid prototyping of code customized for the experiment. The GUI is based on the PyQt5 library.

The Experiment class can also be accessed through the command line interface, bypassing the GUI for more advanced users. It communicates with the main controller via serial port and includes following features:

- A default dictionary of settings containing the name of the experiment, the number of blocks per day and in total, the number of trials in a block, the number of animals, the Arduino serial port address, three time limit thresholds also containing the number of pellets to reward for each threshold, as well as an upload settings function, which sends the parameters to master Arduino.
- Save/load experiment settings and data (serialized data structure to write to a file efficiently).
- The randomized stimulus presentation sequence is generated in the form of a list. The randomization can be modified to adhere to specific experiment requirements. For example, to limit the number of times the same stimulus can be presented in succession.
- A function to run a block of trials for a given list of randomized locations.
- A function to register the initiation of a trial and its outcome (which reward location was chosen in response to the cue). This information is immediately saved to a text file and into the class data container.
- A function to administer additional reward or cancel a trial at any moment.
- The possibility to add a comment to a trial.

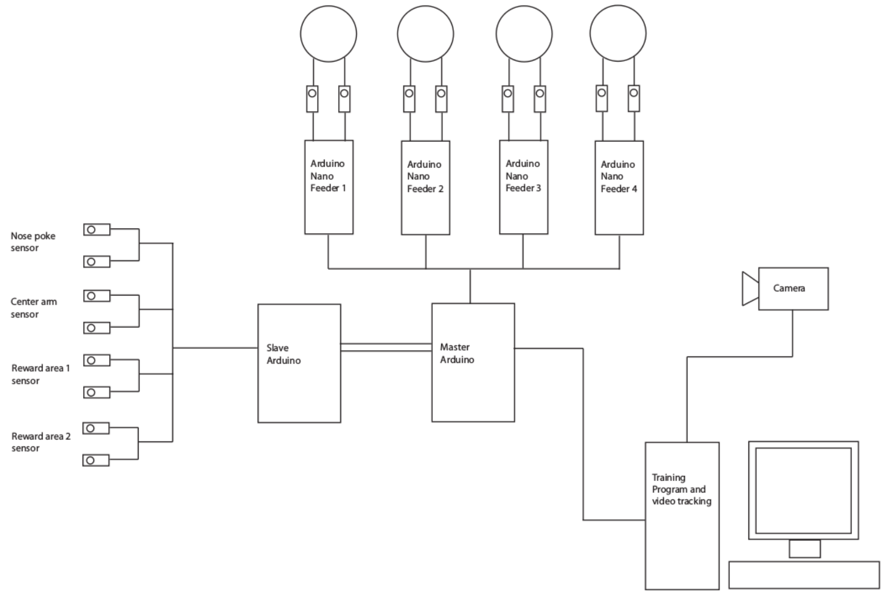

Supplementary Figure 2: Hardware schema showing the computer running the training software and video tracking, the camera recording the trials, the main microcontroller acting as a master and connected to the four feeder units, each controlled and monitored by their own microcontroller, and also connected to another microcontroller that registers the input from the four infrared beam sensors pairs.

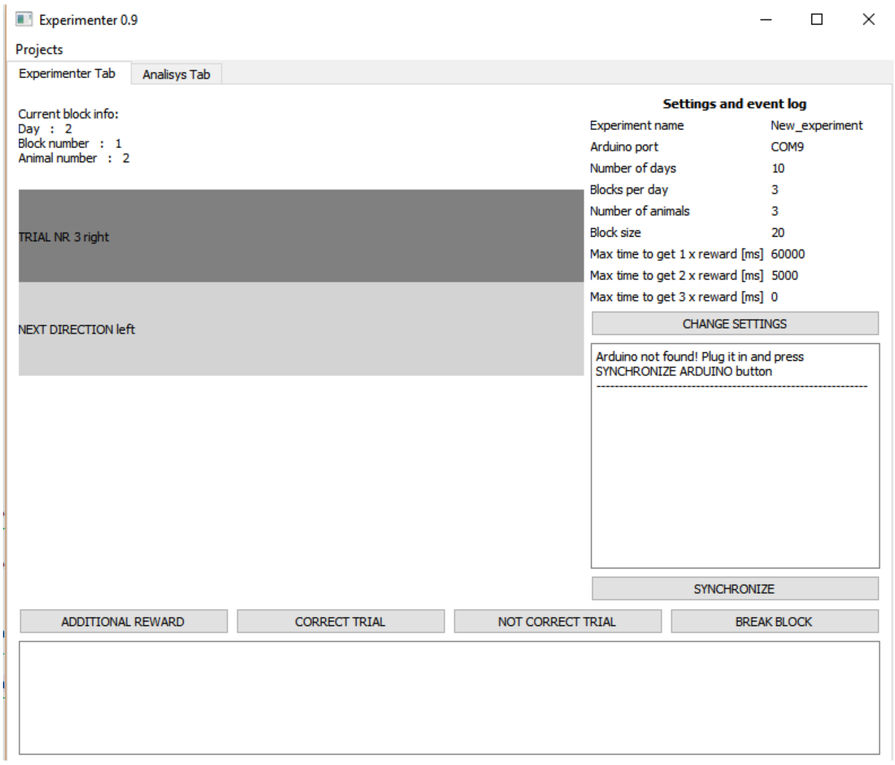

Supplementary Figure 3: Screenshot of the software program showing the information regarding trail in progress including the training day, the block number, and the number identifying the animal. The current trial is shown underneath this information in a rectangle colored according to the rewarded side. A preview of the next trial is shown in the rectangle underneath. The experiment settings are shown in the left hand column including the name of the experiment, the name of the connected microcontroller, the blocks per day, the number of animals, the number of trials per block, and the maximum time allowed defining the three response windows. The 'change settings' button directly underneath allows the user to adjust these settings. The window below displays feedback received from the microcontroller and the button below this window allows the user to manually synchronize the microcontroller in case the automatic synchronization experiences problems. The four buttons spanning across the screen horizontally facilitate manual control of the maze including the ability to dispense extra pellets, mark a trial as correct or incorrect, and end the block, in case the animal has lost interest and is no longer initiating trials. In the area below the user can write notes regarding any unusual events or comments regarding the trial if necessary.

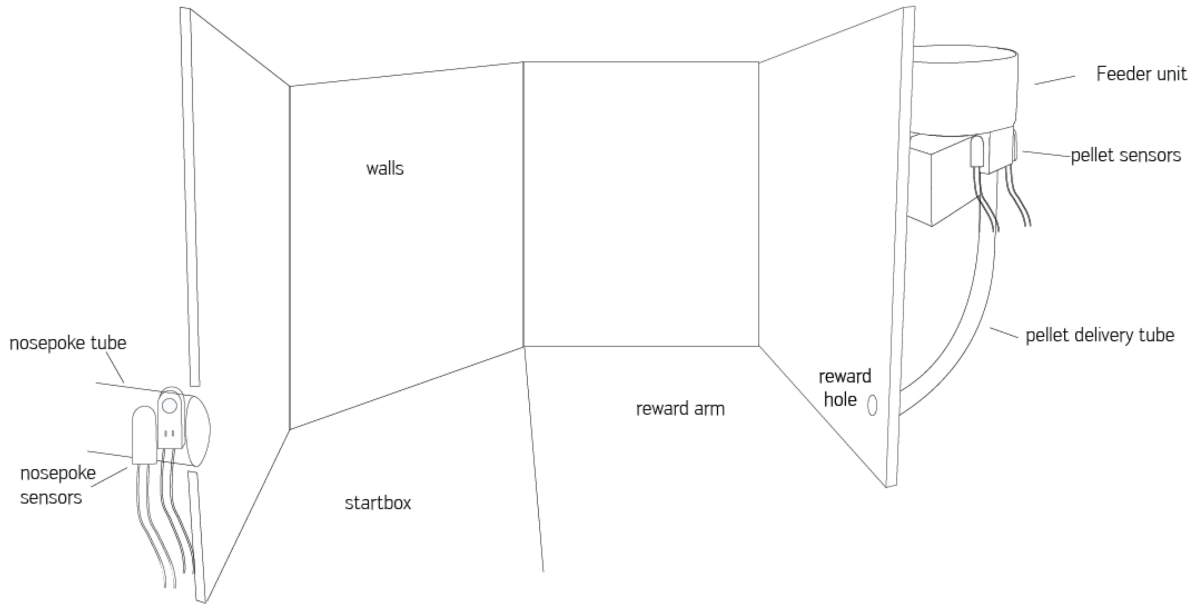

Supplementary Figure 4: Cross-section of the maze set up in the first phase including 40 cm high walls, nose poke tube with infrared beam sensors. The reward arm positioned immediately against the startbox and the feeder unit behind the wall consisting of a pellet basin connected to a servomotor and a pellet delivery tube, which delivers the pellets to the animal in the maze.

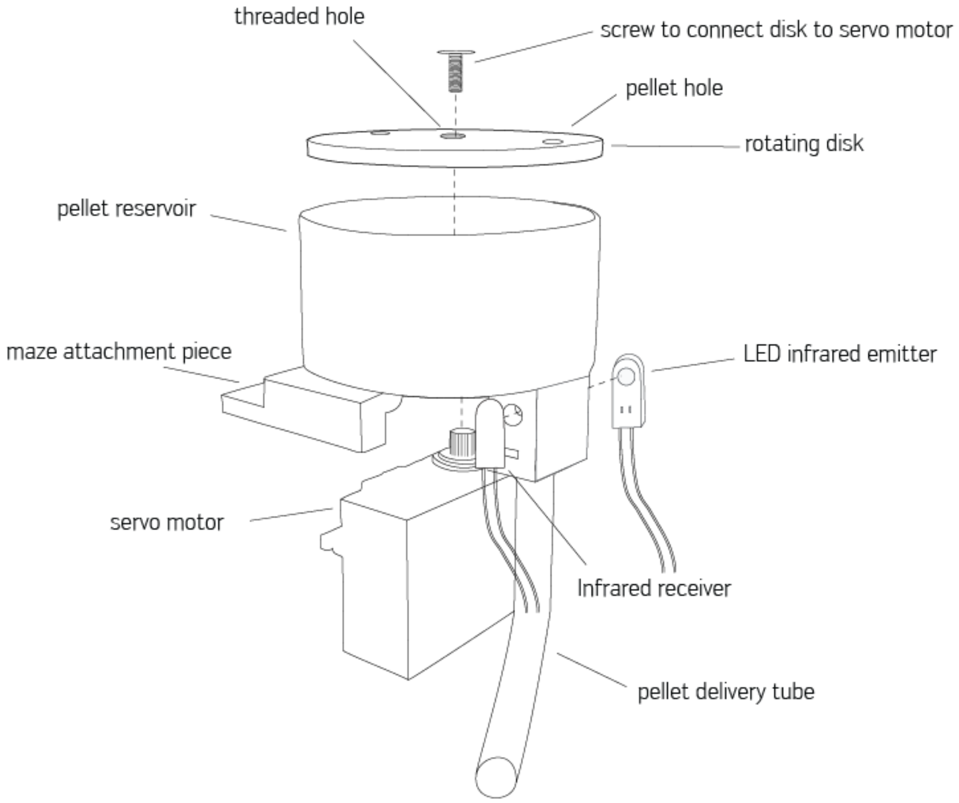

Supplementary Figure 5: An extruding piece of the pellet reservoir containing two holes for screws allows the feeder unit to be attached to the maze. Pellets are dispensed by means of a rotating disk containing two pellet holes connected to the servomotor with a screw. The bottom of the pellet reservoir contains one hole, which, when vertically aligned with the hole on the disk allows pellets to drop into the tube below. The infrared beam created between the LED infrared emitter and receiver detects pellets dispensed into the delivery tube. The microcontroller attached to the feeder unit receives the number of pellets to be dispensed from the main microcontroller and spins the disk until the sensor counts indicate that this number has been reached.

Randomization

The sequence of cues across trials is crucial for successful task learning. A completely random sequence will most likely result in a negative learning outcome as rats are sensitive to spurious correlations and runs in the trial sequence. Especially in the early stages of learning, rats will respond to perceived patterns in the sequence of cues and reward deliveries. The difficulty is compounded by the fact that in a binary task, a random strategy will lead to reward in 50% of the trials, which may quell motivation for learning the strategy leading to 100% rewards. One of the most common strategies applied by rats in a two-alternative forced-choice task is known as alternation, which involves alternating between the possible reward areas. This strategy may reflect natural foraging behavior where it is not strategic for the animal to return to a depleted food source [2]. The randomization of the trials must be designed to discourage this natural tendency to alternate. It must, however, also not allow the same side to be rewarded too many times in a row since this can result in the development of a bias to the frequently rewarded side. Once such a bias is established it can persist for several sessions and prevent the animal from learning the task. It is therefore crucial to test the randomization algorithm for repetitive patterns and the amount it alternates between sides, which can encourage alternation behavior in the animals.

Randomized sequences of stimulus presentation are therefore tested for patterns that may establish a bias either towards one particular side or towards alternation behavior. Sequences are generated at the start of each block. When a sequence does not meet the requirements it is discarded and a new sequence is generated and tested against the requirements.

To examine possible patterns occurring in the generated sequences that meet the requirements the randomization is run many times and the generated sequences analyzed for potential patterns. Here the randomization has been created ten thousand times to observe the frequency in which certain patterns occur within a block of 20 trials.

The number of left and right trials within a block must be balanced. The figure below displays the distributions of the number of trials rewarded on the right side of the maze, and the number those rewarded on left. These distributions must be almost identical to avoid biases. When many consecutive trials reward a particular side, a bias towards that side may be formed. To avoid such a bias the randomization cannot contain more than three trials in a row of a particular side. However this restraint can result in sequences that contain a relatively high amount of alternation transitions between trials (see figure on the right), encouraging alternation behavior. Reducing the number of allowed alternation transitions corrects for the natural tendency of the animal to alternate. The randomization simulation resulting from the implementation of this measure does not allow for more than 9 alternation transitions in a block of 20 trials, shown in the figure below. The amount of left to right versus right to left transitions should also be equivalent. The number of trials per block varies per phase. These checks should therefore be performed for all block sizes used.

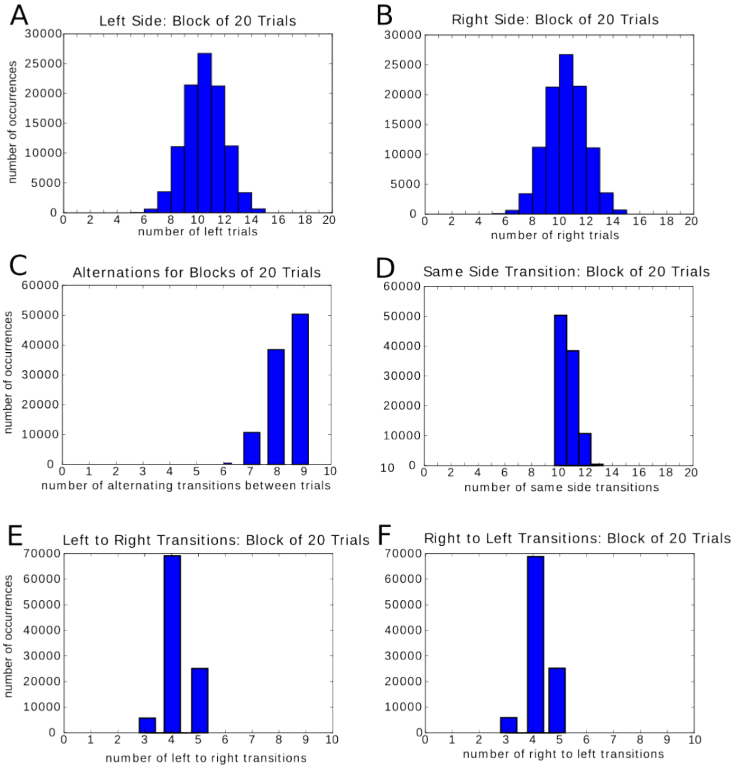

Supplementary Figure 6: Testing the randomization for patterns. Randomization for blocks of 20 trials simulated 100,000 times. The number of occurrences where the phenomenon mentioned in the title (for instance: 'trial is left', or 'trial side is identical to previous side in previous trial' (same side transition)) occurred in each simulated block is shown on the y-axis. The distributions of the amount of left and right trials occurring in a block are identical to prevent any biases from developing. With two possible choices the transition from one trial to the next can either be to the same side or to the opposite side. The likelihood of the next trial being to the opposite side should be slightly lower than the chance that it is to the same side in order to prevent the animals from alternating between locations on every trial, a common bias seen in rats. Distributions of left to right and right to left transitions should be identical in order to prevent bias forming.

Strategy Simulations

As mentioned previously rats are excellent in identifying patterns in the randomization in order to predict which side has a high probability of delivering reward in the upcoming trial, given the outcomes of the previous trials [3]. Predictable patterns in the randomization must be avoided as they may be learned by the animal, providing a way to solve the task without attending to the cue, obtaining the reward significantly more than 50% of the time and minimizing effort. Such behavior would hinder the learning of the intended task.

Thus, the use of common strategies (e.g. alternation) must be discouraged by ensuring that these do not result in an excess of obtained rewards. The threshold at which an animal determines that a strategy is viable differs per animal. However as a general rule the randomization should not reward these strategies more 50 percent of the time. Our pilot studies revealed that when adherence to a strategy consistently results in reward over 60 percent of the time animals are likely to use a strategy. This was especially true for common strategies requiring limited mental resources such as alternation.

Several common behavioral strategies related to patterns in the randomization have been observed in pilot data. Common simple strategies are alternation, or always choosing one particular side. Slightly more complicated are patterns such as left, left, right, or right, right, left, etc. More sophisticated strategies involve using success of a previous decision to determine the next choice of side. For instance an animal that systematically returns to the same location if it was rewarded, and switches to the opposite location when not rewarded is using what is often referred to as a 'win-stay' strategy. A 'win-shift' strategy involves choosing the opposite location to the one rewarded in the previous trial.

The randomization must not reward these strategies either and should therefore be checked against patterns that may encourage this behavior. Scoring the success of common strategies when applied to the generated randomizations can help identify patterns present in the presentational sequence of the stimuli.

First, simple strategies were simulated, meaning static strategies that are not based on changes in the environment such as the tone, or whether a reward was given at a particular location in a previous trial. Such strategies include consistently choosing only the left or only the right side, alternating between the two sides, or choosing sides based on a set pattern, such as go left twice, then right once, then left twice again, etc. None of these strategies will reward the rat more than 50 percent on average as can be observed in Supplementary Figure 7.

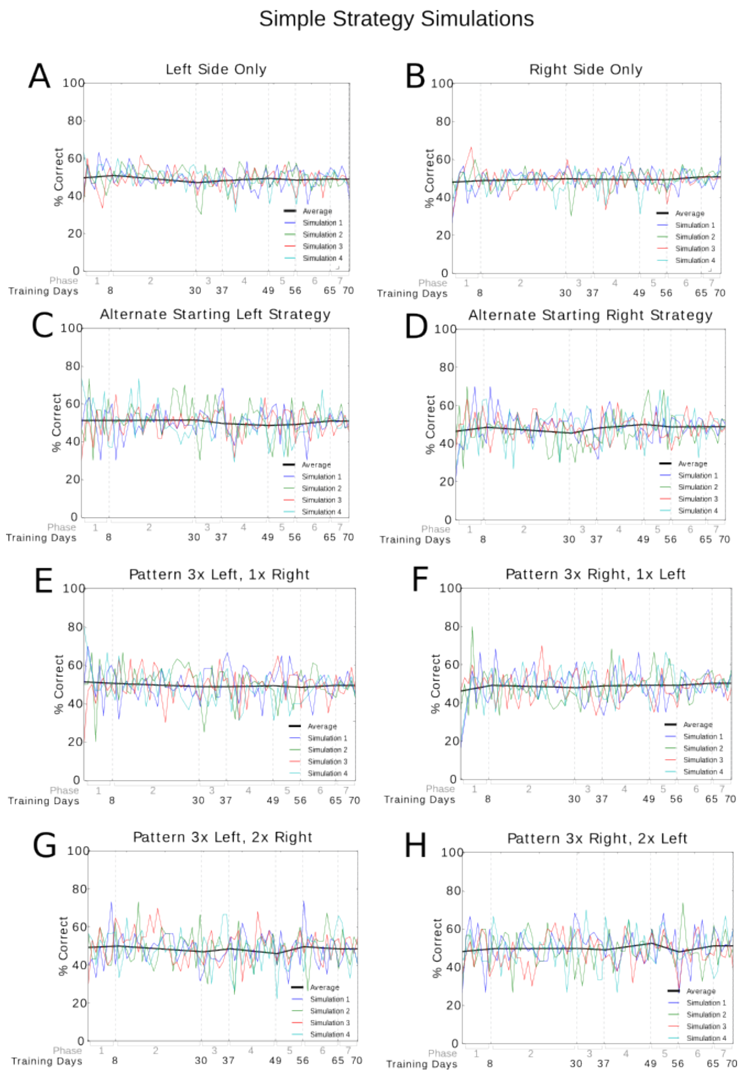

Supplementary Figure 7:
Simple strategy simulations. Simulated rats choosing sides according to a predetermined strategy are presented with sequences of cues generated by the same randomization sequence used in the experiment. The percentage of correct choices (y-axis) is shown for each training day (x-axis). None of the simulated strategies result in a score higher than 55% on average.

A slightly more complex strategy involves responding to a change in the cue tone. In this case the animal switches the chosen reward location when the cue tone has changed from one trial to the next. The use of this strategy indicates that a change in tone between trials is associated with a change of reward location instead of associating a specific tone with a particular side. This strategy can result in a run of successful trials if the animal happens to start on the side correctly associated with the tone. An animal displaying this behavior may appear to have learned the task of associating a particular tone with a specific reward location, when in fact, it has learned a different task, namely to switch to the opposite reward location on a change of tone. On the other hand, an incorrect initial decision in the first trial will result in a block of trials where the animal will not receive any rewards unless a strategy-change occurs within the block of trials. It is also possible for the animal to start out correctly but become distracted during the course of the block and miss a tone change, after which the outcome of the strategy will reverse.

Simulating this switch-on-tone-change strategy did not result in success more than 50% of the time on average. This included simulations in which the simulated rat was distracted (random side was chosen) every 5 or 10 trials, or a random number of trials between 5 and 10 each time, after which the tone change strategy was continued, now based on the random response from a 'distracted' trial.

The choices of the animals were analyzed for compliance with common strategies during the course of the experiment. We observed that the natural tendency of the animals towards alternation between reward areas was remarkably strong. A reward percentage of chance level did not deter the animals from regressing to the use of this strategy relatively frequently, even in later stages of training. Consequently the randomization was adjusted to discourage this tendency in the full task (phases 5 to 7) by increasing the chance that a particular location was rewarded twice in a row to 60 percent, thereby lowering the chance of reward at the opposite location in the next trial to 40 percent. This proved sufficient to break the habitual alternation.

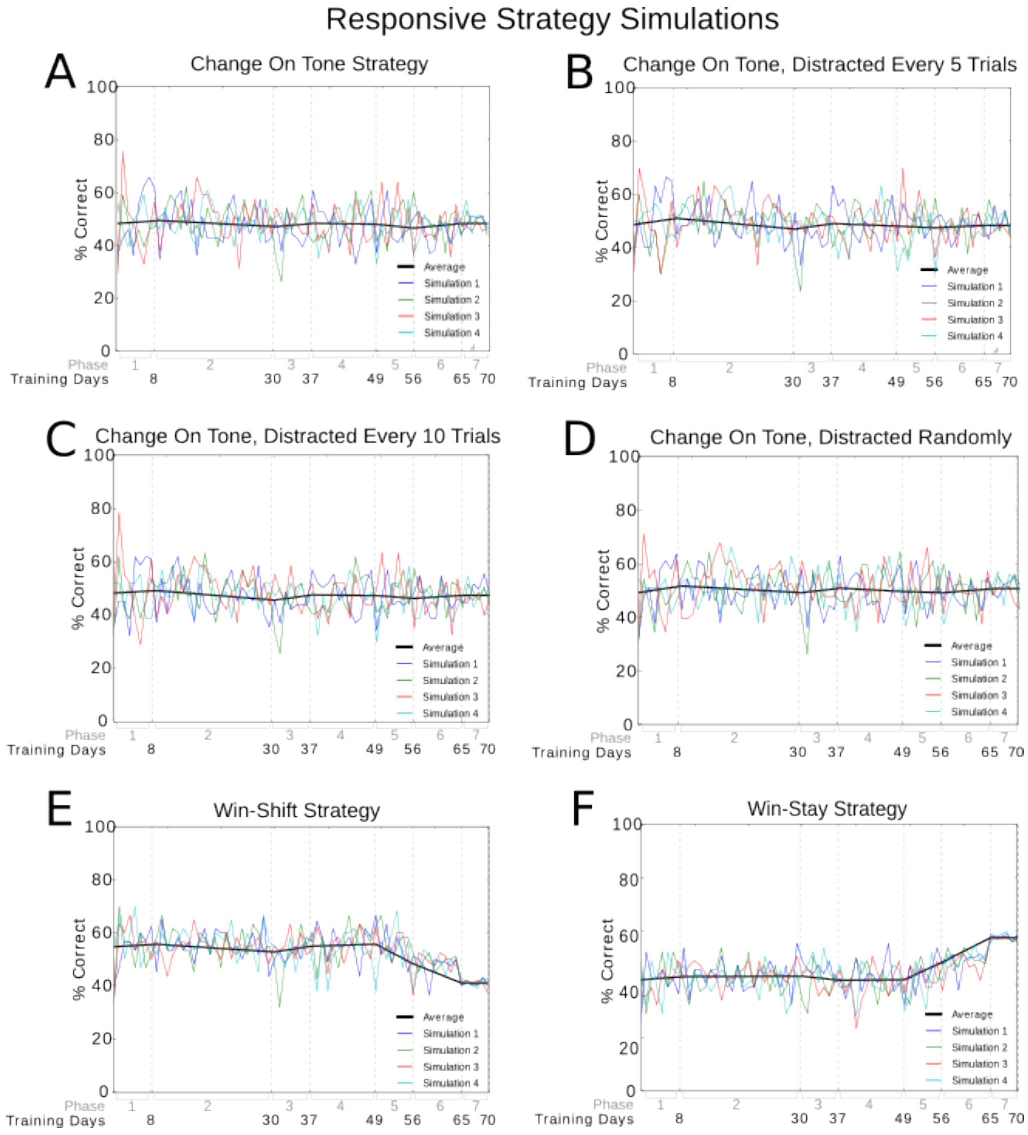

Supplementary Figure 8: Responsive strategy simulations. The percentage of correct choices (y-axis) is shown for each training day (x-axis). Simulations responded to sequences of cues, generated with the same algorithm used in the experiment, according to a particular strategy. The simulations were programmed to change their choice in side in response to a change in the cue tone (with several variations such as 'distractions' occurring every 5 or 10 trials, or at a random number between 5 and 10.) The win-shift and win-stay strategy simulations were programmed to change sides in relation to their success in the previous trial. In the win-shift simulation this meant that if the choice the simulation made in the previous trial was correct then the opposite side was chosen in the next trial, and an incorrect trial resulted in a choice for the same side as chosen in the previous trial. For win-stay the same side was chosen again following a correct trial, and the opposite following an incorrect trial. These two strategies disregard the cue tone altogether. None of the above strategies resulted in a score higher than 60%.

Animal Scores on Use of Strategy

The simulations of the various strategies discussed earlier indicate that the animals could not use one of these strategies and score above 60 percent. To ensure that the animals did learn the task and were not applying a strategy the response of the animals for each trial was evaluated based on its response in the previous trial. That is, the response of the animal is compared to the manner in which it would have responded if it had behaved in accordance with a predetermined strategy.

Scoring the reaction of each of the strategy stimulations on the choices made by the animal in the previous trial reveals a similar pattern as was seen in the strategy simulations previously. The figures show the percent of correct choices the animals made during the experiment that would have also been correct if they had acted according to a win-stay, win-shift, or alternation strategy. Considering the actual scores are much higher we can conclude that animals could not have relied solely on one of these strategies to achieve these results.

Training Methods (protocols)

Animals

Four 6 months old Long Evans male rats were housed in pairs in large cages (610 x 435 x 215mm) maintained on a reversed 24h light/dark cycle, and food deprived to 85% of their ad libitum weight, based on a ad-libitum feeding weight curve by animal supplier Janvier. Training sessions took place during the dark portion of the cycle.

Food Restriction and Feeding

In order to learn the rats must complete at least 30 trials per day, the rats must be motivated and food deprived to 85% of their ad libitum weight. Food intake was restricted to 15 grams per day for each rat, of which on average 10 grams were earned as reward in the maze during the task, and the remaining food (15 grams minus reward earned in maze) fed in the home cage. In group housed animals however, this may be problematic, as the dominant rat will have a tendency to consume more food, leading to a decrease in motivation the following day and an insufficient amount of food available for the non-dominant animal. Possible solutions are to place the pellets in opposite corners of the cage enabling animals to consume the pellets at a safe distance from each other, or to feed the animals separately. The latter, however, requires habituating the animals to separate cages for feeding. For optimal results additional blocks of trials should be added per training day, with rest periods between blocks so that the entire daily nutritional need of 15 grams is rewarded during the task.

Pellets fed as rewards were 45mg Supreme Mini-Treats from Bio-Serv. In this particular experiment a mix of bacon and apple flavors was used, however any flavor, or non-flavored pellets also suffice [4]. Larger pellets are not recommended however as they decrease the amount of trials that can be run daily.

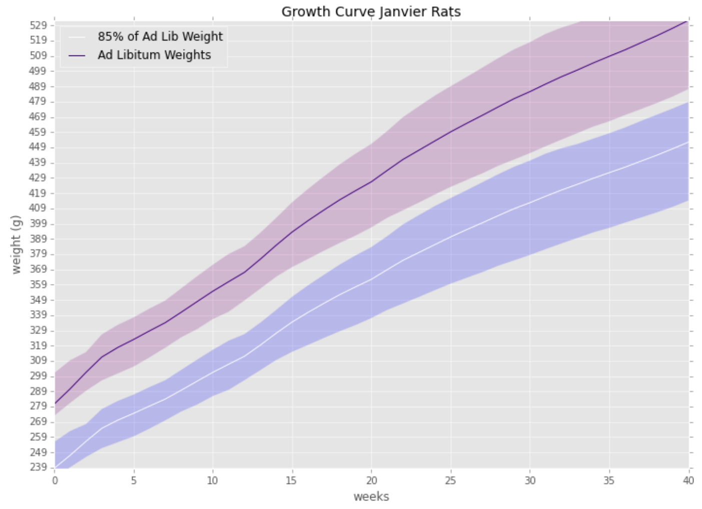

Supplementary Figure 9: Growth curve showing the weight in grams on the y-axis and the age in weeks on the x -axis. Data were taken from average weight per week of age provided by the supplier Janvier and supplemented by our own data from eight animals from Janvier previously used in experiments for weeks 25 to 40 as Janvier could not provide weights for this age range. Animals were weighed three times per week. These weights were averaged and plotted per animal on this growth curve chart weekly. Weights should fall within the purple shaded error band around the 85% curve. When this was not the case the home cage feeding was adjusted accordingly.

Task Design

Trials are grouped in blocks. The number of trials per block varies per phase. A rat remains in the maze for the duration of a block and is then placed back in the home cage. Meanwhile the maze is cleaned, and another rat is placed in the maze. This block design allows animals to rest, drink, and regain motivation in between blocks. Training with four animals per group creates a sufficiently long recovery time for the animals to regain motivation before entering the maze again for the next block. In this experiment trials were divided into four blocks of trials per day. Initially the animals may still be wary of the new environment and easily distracted thus the blocks in the first phase should consist of 10 trials only in order to provide sufficient pauses in the training. As the animals become more at ease in the environment and accustomed to the task and daily routine the amount of trials increases to 15 per block in Phase 2, then 20 trials per block, and finally 25 trials per block.

When pair housed one animal may be dominant over the other. This dominance is often displayed in the form of the dominant animal pinning down its cage mate on its back. It is best to always take the dominant animal first as natural rat behavior dictates the dominant animal to have priority in exploration and access to food [5]. If the non-dominant animal is placed in the maze before the dominant animal, the dominant animal may act aggressively upon his return to the home cage. In experimental designs that do not require counterbalancing for this aspect, placing animals in the maze in the same order everyday will help them establish a routine, which may help reduce stress.

Monitoring performance

Performance was monitored daily. At the end of a training day the performance of each rat was plotted and compared to previous days. In this task thresholds to obtain reward are altered incrementally. As the requirements for obtaining a reward becoming increasingly more demanding it is to be expected that performance may decrease

somewhat with a change in requirements. For some animals however the increased difficulty may result in a drop in obtained reward, and engagement in the task. Because of this, the previously required behavior was rewarded, though with fewer pellets. For instance for the previously desired behavior reward two pellets, and for the newly desired behavior reward four. We found that even an increase by one pellet for 'better' behavior will positively bias the rats towards displaying that behavior more often. Dominant animals may, in response to receiving fewer pellets, choose to apply an easy strategy such as alternation [6], or exhibit decreased motivation. In those cases, we avoided feeding animals in the home cage where one animal may eat more than the other, and all food was provided during the task, ensuring animals consumed an equal amount of food each day. Alternatively, the threshold may be lowered temporarily to such a degree that the rat is capable of achieving it, to then be slowly be increased in difficulty again.

The reaction time was also used to determine the amount of reward. The standard reward for a correct choice within the time limit was two 45 mg pellets. One extra pellet was given for reaction times within three seconds. This time limit was based on observation. Longer reaction times often indicated hesitant behavior or lack of attention. In this manner, purposeful and goal directed behavior was encouraged. In the first two stages an extra pellet was also rewarded if the animals exceeded their criteria for reward. For instance, if obtaining a reward required a 30-degree turn, then a 60-degree turn was rewarded with an extra pellet.

The first and second phase of the cue training included 'hint' trials where one reward was given immediately following the cue tone. On the first day of the first phase all trials consisted of hint trials. The number of hint trials was gradually decreased over several days. Near the end of phase two hint trials were rarely used. After these first two phases hints were only given occasionally to improve motivation. These hint trials were excluded in the manual and reward based scores.

Analysis Methods

To test the accuracy of automatic scoring, all trials were scored both manually and automatically. Manually each trial was scored as correct, incorrect, or canceled. A trial was invalid or canceled if the animal did not react to the cue within the time limit or the animal did not leave the start-box before the time limit was reached, in case of hardware or software malfunction, for instance if the cue was not given, or due to human error (if, for example, the researcher interfered with the trial accidently), or if a hint was provided in the form of one pellet dispensed from the reward area immediately following the tone. This was necessary at times to calm an anxious animal, or to motivate an unmotivated animal. For number of valid trials per day see Supplementary Figure 11.

For each trial the training program records which side was cued, which reward area sensors the rat activated first, the time between the activation of the nose poke sensors

and the activation of the reward area sensors (reaction time), how many pellets were rewarded by the computer, and how many extra pellets were rewarded manually by the operator.

Several different scoring methods were tested and compared. The first consisting of summing the trials marked manually as correct. Also, the trial information gathered by the software was used to compare the cue side with the reward area sensors that were activated first. If these were identical then the trial was marked as correct, if they differed the trial was marked as incorrect, and if the trial was marked as not valid then the trial was not taken into consideration for scoring. The time limit within which a trial was valid varied per phase (see Supplementary Table 1).

The compact size of the maze during the first phases presented a problem, as it was not uncommon for the tail of the rat to activate the sensors. Consequently, sensor information was not always reliable in the first four phases. To score the automatically gathered trial data correctly without relying on the sensor activation required examining the rewards given. In phase 1, the scoring criteria for a correct trial were three or more rewarded pellets. This served not only to motivate the animals but also for feeding purposes, to ensure they received the majority of their daily food intake in the maze, as the first phase consisted of only 30 trials per day. In phase 2 to 5 the criteria consisted of two or more pellets, and in phase 6 and 7 one or more pellets.

Not all training days contained an equal amount of valid trails per day. Trials could be declared invalid for reasons such as the occurrence of a technical error, or the inadvertent activation of the nose-poke sensors. Supplementary Figure 11 provides an overview of the amount of valid trials per day for each animal (average over animals in black).

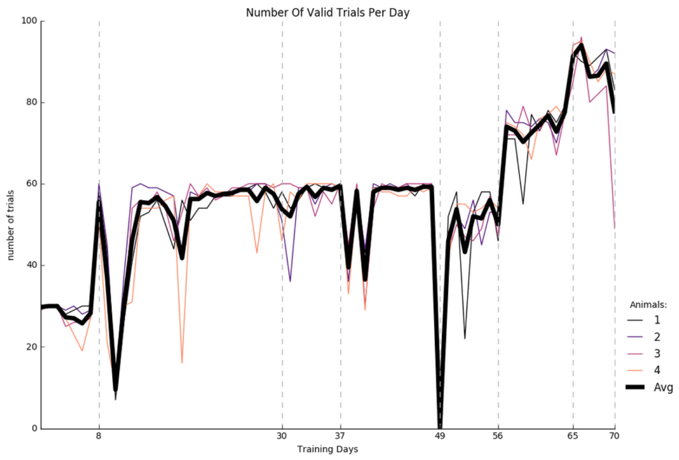

Supplementary Figure 10: Number of valid trials (y-axis) per day (x-axis). The large dip at day 49 was due to a hardware failure due to which no training could take place that day.

Inter-trial Intervals

The inter-trial intervals were determined through a video analysis where the location of the animal in the maze and its direction of movement were tracked. The sampling speed of the video was 30 frames per second. The inter-trial interval was defined as the time in seconds between the moment the animal moved away from the reward area after a valid trial and the subsequent initiation of a new trial. Valid trials were defined as a sequence of video frames where the animal is stationary in the start box facing the nose-poke (south), then proceeds to move north up the central arm, and enters one of the reward areas and is stationary there for at least 1 frame (33.33ms). The exact parameters determining the direction of movement and minimal amount of time spent in the start box and central arm qualify a valid trial varied per phase according to the maze configuration. See Supplementary Table 2 below.

	**Minimum time stationary in start box**	**Direction of movement sequence in start box**	**Minimum northbound time in central arm**	**Direction of movement in central arm**	**Minimum time between trials**
**Phase 1 to 4**	33ms (1 frame)	south, stationary, then north, northeast or northwest	none required	none required	1s (30 frames)
**Phase 5**	333ms (10 frames)	south, stationary, then north	100ms (3 frames)	north	1.5s (45 frames)
**Phase 6**	333ms (10 frames)	south, stationary, then north	367ms (11 frames)	north	2s (60 frames)
**Phase 7**	333ms (10 frames)	south, stationary then north	367ms (11 frames)	north	2s (60 frames)

Supplementary Table 2: Parameters for each phase to identify valid trials.

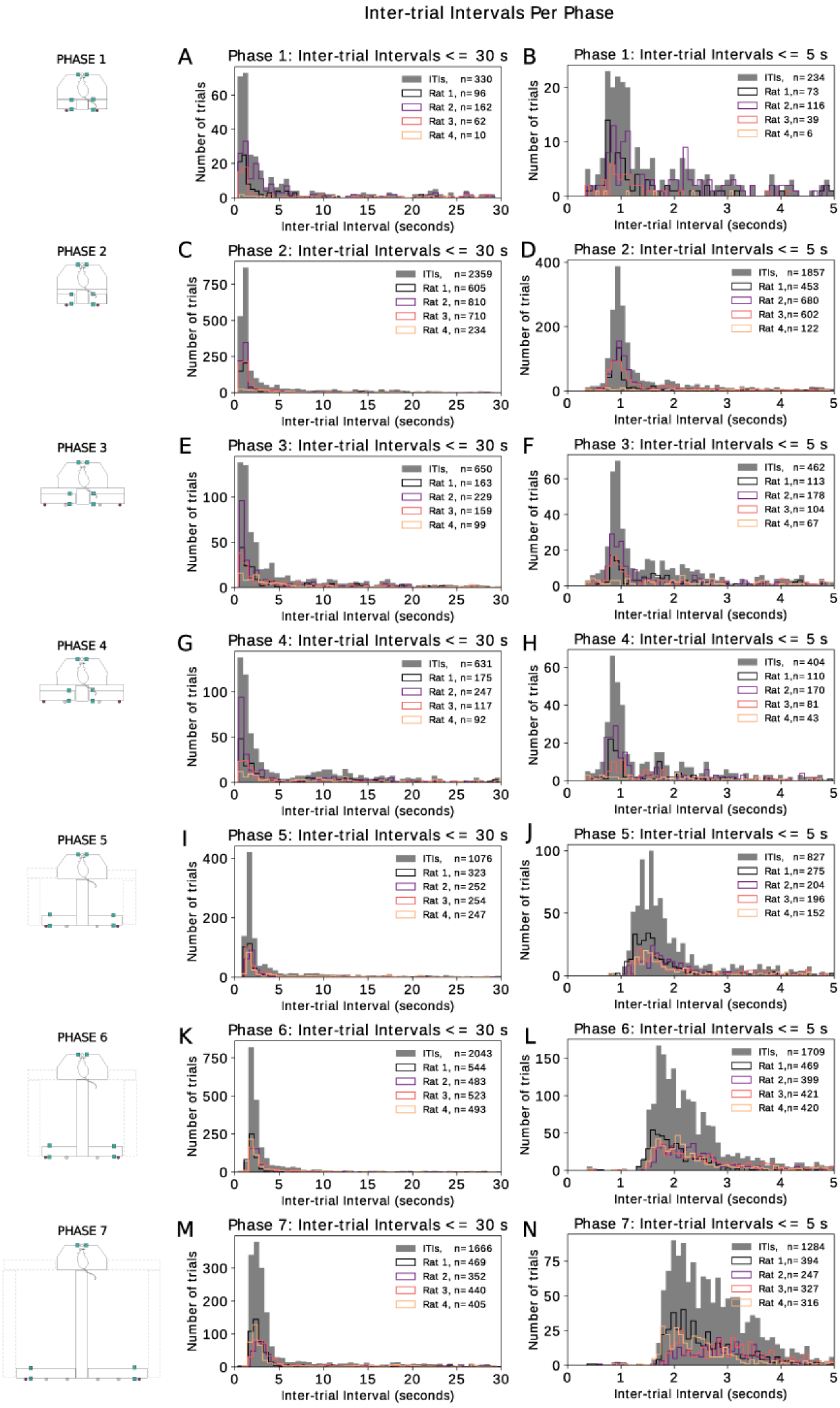

Supplementary Figure 11: Inter-trial intervals per phase, where the number of trials is shown per inter-trial interval bin. The figures on the left show inter-trial intervals smaller or equal to 30 seconds distributed over 50 bins, and those on the right zoom in to only those smaller or equal to 5 seconds, also divided into 50 bins, to better view the shape of the distribution. The grey histogram represents the inter-trial intervals from all animals. Inter-trial intervals per animal are shown in colors black, purple, pink, and yellow for animals 1 through 4 respectively. The total number of trials shown in the figures is visible in the first row of the legend, while the total number of trials per animal for the data shown in the figures is shown to the right of the animal number in the legend.

The Python and Arduino code necessary to run an experiment using the training method described in this paper can be found at:
https://github.com/estherholleman/AutoTrainProgram

Supplementary References

1. Modlinsk, K., Stryjek, R., Pisula, W. Food neophobia in wild and laboratory rats (multi-strain comparison). Behavioural Processes 113, 41-50 (2015).

2. Sanderson, D.J., Bannerman, D.M. The role of habituation in hippocampus-dependent spatial working memory tasks: evidence from GluA1 AMPA receptor subunit knockout mice. Hippocampus 22, 981–94 (2012).

3. Fam, J., Westbrook, F., Arabzadeh, E. Dynamics of pre- and post-choice behaviour: rats approximate optimal strategy in a discrete-trial decision task. Proc R Soc B 282, https://doi.org/10.1098/rspb.2014.2963 (2015).

4. Powell, N.J., Redish, A.D. Representational changes of latent strategies in rat medial prefrontal cortex precede changes in behavior. Nature Communications 7, https://doi.org/10.1038/ncomms12830 (2016).

5. Barnett, S. A. The Rat: A Study in Behavior. (Aldine Transaction Publishers, 1963).

6. Lester, D. Exploratory behavior of dominant and submissive rats. Psychon Sci 9: 285. https://doi.org/10.3758/BF03332224 (1967).
